# Controls of H_2_S, Fe^2 +^, and Mn^2 +^ on Microbial NO_3_^–^-Reducing Processes in Sediments of an Eutrophic Lake

**DOI:** 10.3389/fmicb.2020.01158

**Published:** 2020-06-16

**Authors:** Adeline N. Y. Cojean, Moritz F. Lehmann, Elizabeth K. Robertson, Bo Thamdrup, Jakob Zopfi

**Affiliations:** ^1^Department of Environmental Sciences, Aquatic and Stable Isotope Biogeochemistry, University of Basel, Basel, Switzerland; ^2^Department of Marine Science, University of Gothenburg, Gothenburg, Sweden; ^3^Department of Biology and Nordic Center for Earth Evolution, University of Southern Denmark, Odense, Denmark

**Keywords:** NO_3_^–^ reduction, N-processes partitioning, denitrification, DNRA, inorganic electron donors, lake sediments, eutrophic lake

## Abstract

Understanding the biogeochemical controls on the partitioning between nitrogen (N) removal through denitrification and anaerobic ammonium oxidation (anammox), and N recycling via dissimilatory nitrate (NO_3_^–^) reduction to ammonium (DNRA) is crucial for constraining lacustrine N budgets. Besides organic carbon, inorganic compounds may serve as electron donors for NO_3_^–^ reduction, yet the significance of lithotrophic NO_3_^–^ reduction in the environment is still poorly understood. Conducting incubation experiments with additions of ^15^N-labeled compounds and reduced inorganic substrates (H_2_S, Fe^2+^, Mn^2+^), we assessed the role of alternative electron donors in regulating the partitioning between the different NO_3_^–^-reducing processes in ferruginous surface sediments of Lake Lugano, Switzerland. In sediment slurry incubations without added inorganic substrates, denitrification and DNRA were the dominant NO_3_^–^-reducing pathways, with DNRA contributing between 31 and 46% to the total NO_3_^–^ reduction. The contribution of anammox was less than 1%. Denitrification rates were stimulated by low to moderate additions of ferrous iron (Fe^2+^ ≤ 258 μM) but almost completely suppressed at higher levels (≥1300 μM). Conversely, DNRA was stimulated only at higher Fe^2+^ concentrations. Dissolved sulfide (H_2_S, i.e., sum of H_2_S, HS^–^ and S^2−^) concentrations up to ∼80 μM, strongly stimulated denitrification, but did not affect DNRA significantly. At higher H_2_S levels (≥125 μM), both processes were inhibited. We were unable to find clear evidence for Mn^2+^-supported lithotrophic NO_3_^–^ reduction. However, at high concentrations (∼500 μM), Mn^2+^ additions inhibited NO_3_^–^ reduction, while it did not affect the balance between the two NO_3_^–^ reduction pathways. Our results provide experimental evidence for chemolithotrophic denitrification or DNRA with Fe^2+^ and H_2_S in the Lake Lugano sediments, and demonstrate that all tested potential electron donors, despite the beneficial effect at low concentrations of some of them, can inhibit NO_3_^–^ reduction at high concentration levels. Our findings thus imply that the concentration of inorganic electron donors in lake sediments can act as an important regulator of both benthic denitrification and DNRA rates, and suggest that they can exert an important control on the relative partitioning between microbial N removal and N retention in lakes.

## Introduction

The water quality of lakes in Switzerland has greatly improved over the last few decades due to the ban of phosphates in laundry detergents, improved wastewater management and modern treatment technologies (Jakob et al., 2002; Zobrist and Reichert, 2006; Zobrist et al., 2018). Phosphate concentrations have largely returned to pre-eutrophication levels, yet reactive nitrogen levels in Swiss lakes are still relatively high, likely due to continued inputs from agriculture (Zobrist and Reichert, 2006). Lake sediments are hot spots of N transformations and play an important role in the remediation of excess reactive N inputs through nitrate (NO_3_^–^)-reducing processes (e.g., Wenk et al., 2014). Efficient NO_3_^–^ elimination in lakes is mainly due to denitrification, the microbially mediated dissimilatory reduction of nitrate to gaseous N_2_ using organic or inorganic substrates (organotrophic vs. chemolithotrophic denitrification). The anaerobic oxidation of ammonium (anammox) with NO_x_^–^ to N_2_ can also play a role in N removal in lake sediments (Schubert et al., 2006; Wenk et al., 2013; Crowe et al., 2017). Conversely, dissimilatory reduction of nitrate to ammonium (DNRA), results in the retention of reactive N in the environment. The coupling and the partitioning of the different NO_3_^–^-transforming metabolisms will thus determine the ultimate fate of reactive N.

Denitrification and DNRA compete for the same substrates and electron acceptors (NO_2_^–^, NO_3_^–^). In field and laboratory incubations, the ratio of organic carbon (OC) to NO_3_^–^ availability has been shown to control the relative importance of the different N-transforming processes (Kraft et al., 2014; Hardison et al., 2015; Palacin-Lizarbe et al., 2019). For instance, when the OC/NO_3_^–^ ratio is low, NO_3_^–^ is commonly reduced via denitrification. On the other hand, when the OC/NO_3_^–^ ratio is high, DNRA is often the dominant N-reduction pathway (Nizzoli et al., 2010; Yoon et al., 2015; van den Berg et al., 2015, 2016; Chutivisut et al., 2018).

Traditionally, NO_3_^–^ reduction via denitrification and DNRA has been considered as purely organotrophic processes (Tiedje, 1988). Geochemical evidence (Froelich et al., 1979) and the discovery of nitrate-reducing microorganisms using Fe^2+^ as substrate (e.g., Hafenbradl et al., 1996; Straub et al., 1996) stimulated research on the microbiology and biogeochemistry of this novel mode of N-transformation. Culturing studies, as well as sediment incubation experiments provided putative evidence that this process is performed by diverse microbes in a variety of aquatic environments (Table 1). However, bacterial culture experiments with high substrate concentrations (e.g., 2.5–4 mM NO_3_^–^, 10 mM Fe^2+^; Weber et al., 2006b) are not representative for natural conditions. On the other hand, experimental studies with environmentally relevant substrate concentrations did not always investigate the end products of NO_3_^–^ reduction (i.e., N_2_ vs. NH_4_^+^ production; Chakraborty and Picardal, 2013; Laufer et al., 2016). Recent work has shown an increasing contribution of DNRA relative to denitrification with increasing environmental Fe^2+^ concentration (Roberts et al., 2014; Robertson et al., 2016; Robertson and Thamdrup, 2017). Yet, the environmental relevance of Fe^2+^-dependent nitrate transformations is still poorly understood, as is the effect of Fe^2+^ on the partitioning between N-removal by denitrification and N-retention in the case of DNRA. Similarly, knowledge on the potential role of Mn^2+^, which has also been suggested as potential electron donor for chemolitotrophic denitrification (Aller, 1990; Luther et al., 1997), is completely lacking in lacustrine sediments.

**TABLE 1:** Overview of prior experimental studies that have investigated Fe(II) and H_2_S oxidation coupled to nitrate reduction.

**Electron donor**	**Experimental set-up**	**Electron donor conc. (mM)**	**Main N-product**	**Studied ecosystem / organism**	**References**
**Fe^2+^**	Pure culture	3.5	N_2_0 (N_2_ n/d)	*Pseudogulbenkiana* sp.	Chen et al., 2018
		10	N_2_	*Dechlorosoma suillum*	Chaudhuri et al., 2001
		10	n/d	*Acidovorax, Pseudomonas, Paracoccus* sp.	Muehe et al., 2009
		10	n/d	*Azospira* sp.	Mattes et al., 2013
		10	n/d	*Thiobacillus denitrificans* (ATCC 25259)	Beller et al., 2013
	Enrichment culture	0.25	n/d	Stream sediments^a^	Chakraborty and Picardal, 2013
		4	N_2_0, N_2_	Freshwater/marine/brackish sediments^a^	Benz et al., 1998
		4	n/d	Lake sediments^a^	Hauck et al., 2001
		5–6	NH_4_^+^	River sediments^a^	Coby et al., 2011
		6.6–10	N_2_	Town ditches/brackish water lagoon^a^	Straub et al., 1996
		7–8	NH_4_^+^	Wetland sediments^a^	Weber et al., 2006a
		10	n/d	Swine waste lagoon^a^	Lack et al., 2002
	Incubation	0.08–0.15	N_2_, NH_4_^+^	Lake water column	Michiels et al., 2017
		0.49–0.65	NH_4_^+^	Estuarine sediments	Robertson et al., 2016
		0.1–5	NH_4_^+^	Lake sediments	Robertson and Thamdrup, 2017
		2	N_2_	Activated sludge	Nielsen and Nielsen, 1998
		5	NH_4_^+^	Estuarine sediments	Roberts et al., 2014

**H_2_S**	Pure culture	0.001	N_2_O	*Thiobacillus denitrificans* (ATTC 23642)	Sublette and Sylvester, 1987
		0.02	n/d	Sulfuritalea hydrogenivorans	Kojima and Fukui, 2011
		0.04–0.07	NH_4_^+^	*Desulfovibrio, Desulfobulbus* sp.	Dannenberg et al., 1992
		0.05–0.135	NH_4_^+^	Purified *Thioplocca* filaments	Otte et al., 1999
		0.3	N_2_O/N_2_	*Sulfurimonas* sp.	Takai et al., 2006
		5	NH_4_^+^	*Sulfurospirillum deleyianum*	Eisenmann et al., 1995
	Enrichment culture	1	N_2_	Marine tidal sediments^a^	Kraft et al., 2014
		1.5–3	N_2_(NH_4_^+^n/d)	USAB reactor^a^	Campos et al., 2008
		1.5–3	N_2_, NH_4_^+^	Freshwater sludge reactor^a^	Chutivisut et al., 2014
		4–8	n/d	Freshwater stream mud^a^	Kamp et al., 2006
	Incubation	0.002–0.05	N_2_(NH_4_^+^n/d)	Fjord water column	Jensen et al., 2009
		0.01	N_2_	Lake water column	Wenk et al., 2013
		0.05	N_2_0 (N_2_ n/d)	Marine water column	Brettar and Rheinheimer, 1991
		0.1–0.8	n/d	Marine sediments	Schulz et al., 1999
		0–5	N_2_0 (N_2_ n/d)	Lake sediments	Senga et al., 2006
		0.1–5	N_2_	Marine sediments	Bowles et al., 2012
		1	NH_4_^+^	Lake sediments	Brunet and Garcia-Gil, 1996
		1	n/d	Fluidized bed reactor	Cytryn et al., 2005

*^*a*^Origin of the inoculum for pure culture experiments.*

In contrast, the importance of H_2_S as a direct substrate for both denitrification and DNRA is well established (Table 1). In organic-rich lake sediments, sulfate is efficiently reduced to sulfide (Holmer and Storkholm, 2001), which reacts with iron species to sulfur intermediates or FeS (Zopfi et al., 2004). Some nitrate reducers can use both dissolved and particulate forms of reduced sulfur for their metabolism (e.g., Dannenberg et al., 1992; Kamp et al., 2006; Yan et al., 2018). In natural environments, NO_3_^–^ reduction coupled to sulfide oxidation has been studied in water columns (e.g., Jensen et al., 2009; Wenk et al., 2013; Table 1) and sediments (Brunet and Garcia-Gil, 1996; Senga et al., 2006). However, studies on the end products of sulfide-dependent NO_3_^–^ reduction pathways (i.e., N_2_ vs. NH_4_^+^) under environmentally relevant substrate concentrations are still scarce.

In the present study we aimed to assess the role of different potential inorganic electron donors (Fe^2+^, H_2_S, Mn^2+^) in regulating the overall rates and the partitioning between N-removing (denitrification, anammox) and N-recycling (DNRA) processes in the ferruginous sediments of the eutrophic southern basin of Lake Lugano (Switzerland). Previous studies in this seasonally anoxic basin demonstrated that an important portion of the external NO_3_^–^ load is removed through sedimentary denitrification (Wenk et al., 2014). By conducting incubation experiments with ^15^N-labeled substrates and additions of different inorganic electron donors, we determined whether, and to what extent, lithotrophic nitrate reduction is significant under environmentally relevant substrate concentrations.

## Materials and Methods

### Sampling Site

The south alpine Lake Lugano is located on the Swiss/Italian border and is divided by a shallow sill into a permanently stratified northern basin and a eutrophic, monomictic southern basin (Barbieri and Polli, 1992). Using a gravity corer, we collected sediments at two locations in the southern basin: Figino (8°53′37′′E, 45°57′31′′N, 94 m depth) and Melide (8°57′29′′E, 45°56′22′′N, 85 m depth; Supplementary Figure 1). Sampling campaigns took place in 2015 (December), 2016 (March and September) and 2017 (March and June). Depth profiles of water column temperature, conductivity, and oxygen concentrations were obtained by a winch-operated CTD (Idronaut Ocean Seven 316Plus). Bottom water concentration data for NO_3_^–^, NH_4_^+^, total dissolved Fe, Mn, and S were determined within the frame of a long-term monitoring campaign promoted by the International Commission for the Protection of Italian-Swiss Waters (CIPAIS; Commissione Internazionale per la Protezione delle Acque Italiano-Svizzere) on behalf of the Administration of the Canton of Ticino, and were provided by F. Lepori (SUPSI; University of Applied Sciences and Arts of Southern Switzerland).

### Sediment Depth Profiles

Upon return to the laboratory, duplicate sediment cores from each site were sectioned into 1-cm slices in the uppermost 6 cm of the core, and 2-cm slices from 6 to 20 cm depth. Samples were taken for porosity, TOC, particulate Fe^*II*^/Fe^*III*^, total Mn, and dissolved pore water constituents (Fe^2+^, Mn^2+^, H_2_S, SO_4_^2–^, NH_4_^+^, NO_3_^–^, and NO_2_^–^). For porewater collection, sediment sections were rapidly transferred to 50 mL Falcon tubes and centrifuged at 4700 rpm for 10 min. The supernatant was immediately filtered (0.2 μm), and samples (1 mL) for Fe^2+^ and Mn^2+^ were fixed with hydrochloric acid (40 μL 1 M HCl). Samples for H_2_S determination were stabilized with 20 μL aqueous zinc acetate solution (20% w/v) and stored at 6°C until analysis. The remaining filtered porewater was frozen until analysis of all other compounds. Oxygen microprofiles were measured in the laboratory (21°C) using an amperometric microsensor with a tip diameter of 100 μm (Unisense).

### Sediment Slurry Incubations

Seasonal variation of the potential benthic N-transformation processes was investigated through sediment slurry incubation experiments performed at different times of the year (December 2015, March and September 2016). For the preparation of the slurries, fresh surface sediment (upper 2 cm) was homogenized, and aliquots of 1 g sediment were transferred into 120 mL serum bottles and complemented with 80 mL of anoxic (He purged for 45 min) artificial lake water (Smith et al., 2002; Supplementary Table 1). The artificial lake water was free of NO_3_^–^, NO_2_^–^, and NH_4_^+^, reducing the production of N_2_ from NO_x_ present in natural bottom waters. Serum bottles were sealed with blue chlorobutyl-rubber stoppers and crimped before purging with He for 10 min to remove O_2_ and to lower the N_2_ background for further isotopic measurements. Slurries were pre-incubated overnight on a shaker table (80 rpm) at 8°C in the dark to remove any traces of O_2_, which may still have been present after the initial purging. Labeled ^15^N (e.g., Na^15^NO_3_^–^, ^15^NH_4_Cl; ^15^N 99%; Cambridge Isotopes Laboratories; final conc. ∼120 and 71 μM, respectively; Supplementary Table 2) and ^14^N-substrates (e.g., ^14^NO_2_^–^; final conc. ∼34 μM) were added to identify and quantify potential rates of denitrification, DNRA, and anammox (Nielsen, 1992; Thamdrup and Dalsgaard, 2002). During substrate addition and sampling, the slurries were transferred to an anaerobic chamber with N_2_ atmosphere, and were then returned to the shaker table held at 8°C. Gas samples for ^15^N-N_2_ isotope analysis were taken from the headspace at each time point (4 in total) using a 5 mL gas-tight glass syringe (Hamilton). Two milliliters of the gas were transferred to 3 mL Exetainers (Labco) pre-filled with anoxic Milli-Q water. In exchange for the extracted gas sample 2 mL of anoxic Milli-Q water were added to the incubation vials to maintain constant pressure inside. Exetainers with the gas samples were stored upside down at room temperature until isotopic measurement of N_2_. At T_initial_ and T_end_, 6 mL liquid samples were collected, and immediately filtered for nutrient determination and quantification of DNRA rates. The incubation time for each treatment was determined based on preliminary tests and lasted 3–4 days. Liquid samples were kept frozen or acidified with sulfamic acid (40 mM final concentration; Klueglein and Kappler, 2013) until further analysis of dissolved nutrients and metals, respectively. Preliminary tests, during which samples were taken at four time points, consistently showed linear ^15^NH_4_^+^ production over time. We also tested for the adsorption of NH_4_^+^ to sediment material following an adapted procedure of Behrendt et al. (2013). Briefly, sediment slurries were prepared as described above and known quantities of NH_4_Cl were added, corresponding to final concentrations 5, 10, 25, and 50 μM. The percentage of adsorption was calculated from the difference between the target concentration and the actually determined concentration in the supernatant minus the NH_4_^+^ concentration in control slurries without NH_4_^+^ addition. All preparations were done in triplicates.

### Incubation Experiments With Different Electron Donors

To assess the influence of various inorganic electron donors on the mode and regulation of benthic N-transformations, we performed incubation experiments with microbial biomass from fresh sediment. Sediments used were collected in March and June 2017 for Fe^2+^, H_2_S, and Mn^2+^ addition experiments. Through stirring and centrifugation, microbial biomass was separated from the sediment matrix in order to minimize the effect of the sedimentary organic matter, solid-phase iron sulfides and metal-oxyhydroxides. Briefly, the first two centimeters of duplicate sediment cores were sectioned and transferred into a 2-L Erlenmeyer flask with anoxic (He purged) artificial lake water (Supplementary Table 1), which was immediately closed using a thick gray rubber stopper. After intense shaking and stirring, the slurry was transferred into 50 mL Falcon tubes and centrifuged at 300 rpm for 3 min to separate solid-phase particles from detached microbial biomass. The supernatants were pooled, and aliquots of 70 mL were transferred into 120 mL serum bottles (in triplicate) that were then sealed and crimped. The liquid phases were purged with He (10 min) and pre-incubated on a shaker table (80 rpm) in the dark at 8°C. Different volumes of anoxically prepared solutions of FeCl_2_⋅4H_2_O (100 mM), Na_2_S⋅9H_2_O (50 mM), and MnCl_2_⋅4H_2_O (50 mM) were added (Supplementary Table 2). Then the pH was adjusted with anoxic HCl or NaOH (1 M) to the pH of control incubations without added substrate, and kept constant (±0.2 units) for the duration of the experiment. Substrate additions and subsampling of gas or liquid phase was done in an anaerobic chamber, as described above. Samples (1 mL) for Fe^2+^ and Mn^2+^ determination were fixed with 40 mM sulfamic acid (Klueglein and Kappler, 2013). One mL aliquots for H_2_S determination were preserved with 20 μL zinc acetate (20% w/v). The experiment was started with the addition of Na^15^NO_3_^–^ (∼116 ± 11 μM) and lasted 5–7 days (Supplementary Table 2). Incubations took place in the dark, under gentle agitation at 8°C. Given the stark deviation from *in situ* conditions (i.e., the separation of microbial biomass from sediment and modification of the natural solute concentrations), these incubations were not intended to assess absolute benthic N transformation rates that are representative for the natural conditions. Instead, these incubations allowed us to investigate any differential stimulation/inhibition by the tested electron donors within a well-controlled experimental set-up. As a consequence, results below are presented as percent (%) “stimulation” or “inhibition” of a given NO_3_^–^-reducing processes relative to the corresponding controls without additions. Student’s *t*-tests (*P* < 0.05, Excel) were applied to determine significant differences between measured rates.

### ^15^N-Based Rate Measurements

Denitrification and anammox rates were calculated using ^15^N isotope-pairing technique (Nielsen, 1992), through monitoring the production of ^14^N^15^N or ^15^N^15^N (Nielsen, 1992; Thamdrup and Dalsgaard, 2002) using a Delta V Advantage isotope-ratio mass spectrometer (IRMS; Thermo Fisher Scientific) coupled to a gas chromatograph for gas purification. Rates of denitrification and anammox were calculated from the total accumulation of single (^14^N^15^N) and double-labeled ^15^N-N_2_ (^15^N^15^N) over time as determined by linear regression analysis of excess ^14^N^15^N/^14^N^14^N and ^15^N^15^N/^14^N^14^N trends (Supplementary Figure 2). In incubations with ^15^NH_4_^+^ and ^14^NO_2_^–^,

N2⁢⁢a⁢n⁢a⁢m⁢m⁢o⁢x⁢=⁢N14⁢N15×FNH4+

where F_NH4__+_ is the fraction of ^15^N in NH_4_^+^ (Thamdrup and Dalsgaard, 2002). As anammox was insignificant, the following equation was used to quantify denitrification in incubations with ^15^NO_3_^–^,

N2⁢⁢d⁢e⁢n⁢i⁢t⁢r⁢i⁢f⁢i⁢c⁢a⁢t⁢i⁢o⁢n⁢=⁢N14⁢N15+⁢2⁢×⁢N15⁢N15

DNRA rates were quantified by oxidation of NH_4_^+^ to N_2_ using alkaline hypobromite (Risgaard-Petersen et al., 1995), as in Robertson et al. (2016). The produced N_2_ was then analyzed by GC-IRMS as described above. In incubations with ^15^NO_3_^–^, DNRA rates were determined by linear regression of the concentration of ^15^NH_4_^+^ versus time,

NH4⁢D⁢N⁢R⁢A+⁢=⁢N14⁢N15+⁢2⁢×N15⁢N15

^15^NH_4_^+^ standards were prepared in parallel with the samples in order to test the efficiency of the hypobromite oxidation step (recovery typically > 95%).

### Chemical Analyses

Nitrite concentrations were determined colorimetrically according to Hansen and Koroleff (1999). Concentrations of NO_x_^–^ (i.e., NO_3_^–^ + NO_2_^–^) were measured by chemiluminescence detection using a NOx-analyzer (Antek Model 745; Braman and Hendrix, 1989). Nitrate concentrations were then calculated from the difference between NOx- and NO_2_^–^. Ammonium in porewater and incubation experiments was measured generally by suppression-ion chromatography with conductivity detection (940 Professional IC Vario, Metrohm, Switzerland). In treatments with Mn^2+^ additions, NH_4_^+^ was measured spectrophotometrically, due to interferences during the ion-chromatographic separation. Photometric quantification of NH_4_^+^ was done using the indophenol method (Krom, 1980).

Dissolved Fe^2+^ in porewater and incubation samples was measured with ferrozine (Stookey, 1970). Solid-phase reactive Fe^*II*^ and Fe^*III*^ was quantified by extraction in 0.5 M HCl for 1 h according to Jensen and Thamdrup (1993) and subsequent analysis with ferrozine. Manganese concentrations in the acidic sediment extracts (including Mn^*II*^ and Mn^*IV*^) and in Mn^2+^-amended incubations were determined using inductively coupled plasma optical emission spectrometry (ICP-OES; Agilent Technologies 5100).

Dissolved sulfide (H_2_S, i.e., sum of H_2_S, HS^–^, and S^2−^) in the porewater was quantified by the colorimetric methylene blue method (Cline, 1969). Concentrations in slurries were affected by the presence of Fe^2+^ and, hence, the partial removal of the added H_2_S from solution through precipitation as FeS. Therefore, both the added quantity and the measured concentration in the incubations are reported. Porewater chloride and sulfate (SO_4_^2–^) concentrations were quantified by suppressed anion chromatography with conductivity detection (940 Professional IC Vario, Metrohm).

## Results

### Geochemical Setting of Sampling Sites

**FIGURE 1:**
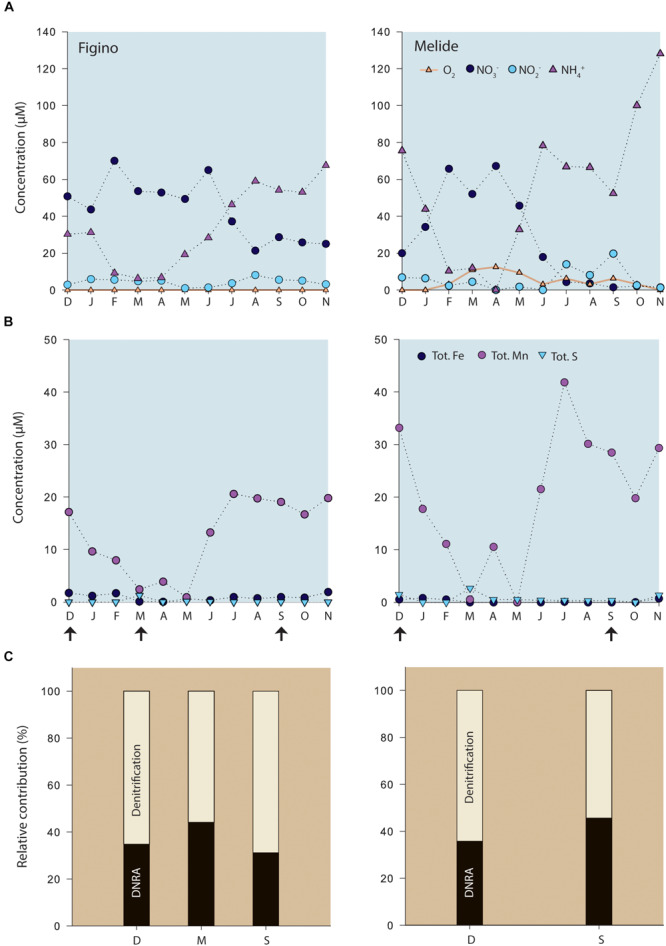
**(A)** Seasonal variation of bottom-water (2 m above sediment surface) concentrations of dissolved oxygen and nitrogen compounds, and **(B)** total dissolved Fe, Mn, and S at the two study locations in the south basin of Lake Lugano (Supplementary Figure 1). No oxygen was detected at Figino during that year. **(C)** Relative contribution of DNRA and denitrification (%) to the total benthic NO_3_^–^ reduction, as determined by sediment slurry incubation experiments at selected time points (arrows). Capital letters at *x*-axes correspond to the first letter of each month from December 2015 to November 2016.

Fine organic-rich (∼8% OC dry mass) sediments were found at both sampling stations in the southern basin of Lake Lugano. High microbial activity within the sediments was reflected by the shallow O_2_ penetration depths of 3.7 ± 0.6 and 2 ± 0.5 mm at Figino and Melide, respectively (*n* = 4, data not shown), as determined by microsensor measurements in the laboratory with oxygenated (∼220–235 μM O_2_) lake water covering the sediment surface. Active bioturbation by higher organisms was not evident, consistent with seasonal bottom-water anoxia for more than 7 months (e.g., Blees et al., 2014). Typically, deep-hypolimnion oxygenation occurs once a year during winter mixing. In 2016, however, the bottom water at Figino remained anoxic throughout the year (Figure 1), whereas at Melide at least low oxygen concentrations were measured throughout most of the annual cycle. The bottom-water concentrations of the different nitrogen compounds varied seasonally as a function of water column stratification conditions. Ammonium concentrations 2 m above the sediments increased during the thermal stratification period at both stations, reaching 68 and 128 μM at Figino and Melide, respectively. With the onset of water-column mixing in February, the downwelling of oxygenated water led to the almost complete oxidation of ammonium and the concomitant production of NO_2_^–^ and NO_3_^–^. The sum of produced NO_2_^–^ and NO_3_^–^ did not match the loss of NH_4_^+^, suggesting that most NO_x_^–^ was further metabolized by respiratory processes in the water column and/or the surface sediments. The bottom water concentrations of NO_3_^–^ varied between 25–65 μM and 1–67 μM at Figino and Melide, respectively, depending on the season (Figure 1). Nitrite concentrations ranged between 0.05 and 14 μM at both stations.

**FIGURE 2:**
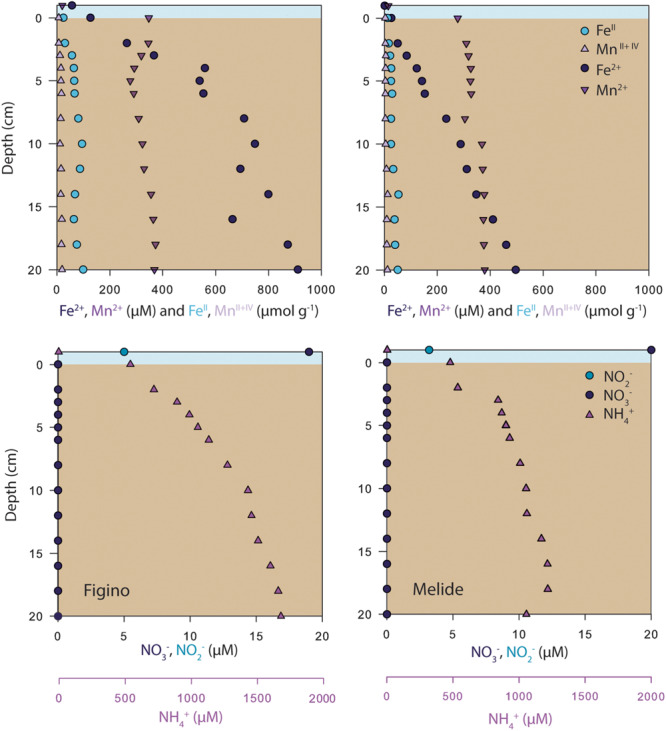
Vertical profiles of porewater solutes (Fe^2+^, Mn^2+^, NO_3_^–^, NO_2_^–^, NH_4_^+^) and particulate phases (Fe^*II*^ and total Mn) in sediments sampled in June 2017 at Figino **(left)** and Melide **(right)**. The bottom water samples were collected 2 m above the surface sediments.

Porewater concentration data of dissolved N compounds (NH_4_^+^, NO_2_^–^, and NO_3_^–^), as well as dissolved and particulate phases of Mn and Fe are shown in Figure 2. At both stations, NH_4_^+^ concentrations near the sediment-water interface were ∼600 μM and increased with depth indicating the active mineralization of organic matter. In the surface sediments, NO_3_^–^ and NO_2_^–^ concentrations were below detection limit (Figure 2), despite the presence of NO_x_ in the bottom waters at the time of sampling (Figure 1), indicating that the sediments represent an efficient sink for these compounds. The porewaters at both stations were further characterized by high concentrations of Mn^2+^ and Fe^2+^. Dissolved Mn^2+^ concentrations were almost identical at both stations (∼350–400 μM, Figure 2) and did not show any strong variation with depth. Concentrations of Fe^2+^ were much higher at Figino, where they increased from ∼150 μM at the sediment-water interface to ∼900 μM at 20 cm depth (Figure 2). Dissolved Fe^2+^ and Mn^2+^ concentrations in the surface sediment layers (0–2 cm) varied throughout the year at both stations, though no clear temporal trends were observed (Cojean, 2019). Particulate Fe^*II*^, Fe^*III*^, and total Mn concentrations were relatively constant with depth, and similar between sites (Figure 2). Free H_2_S was not detected in the porewater at either of the two stations (detection limit 1 μM).

### Benthic N-Transformations

We determined potential rates of benthic denitrification, DNRA, and anammox in anoxic slurry incubation experiments with fresh surface-sediment material, collected at different times of the year (Figure 1). At each site, similar results were obtained for the different sampling months. In ^15^NO_3_^–^ amended slurries, production rates of ^15^N-N_2_ via denitrification were consistently higher than ^15^NH_4_^+^ production through dissimilatory nitrate reduction to ammonium (DNRA). Adsorption of generated ^15^NH_4_^+^ to sediment particles could lead to underestimated DNRA rates. Initial adsorption tests showed, however, that NH_4_^+^ did not bind significantly to the mineral phases in our dilute slurries. Hence, the measured DNRA rates can be considered as not being affected significantly by adsorption artifacts. The contribution of DNRA relative to total nitrate reduction varied seasonally between 31 and 46% (Figure 1). The ratio of denitrification to DNRA (DEN:DNRA) varied accordingly between 1.2 and 2.2. Despite this relatively consistent partitioning between denitrification and DNRA at each station, the absolute potential rates differed markedly across sites. For all sampling dates, except for December 2015, denitrification was higher at Figino than Melide, with measured maximum rates of 85 and 53 nmol N g^–1^ wet sediment d^–1^, respectively. In contrast, DNRA rates were slightly higher at Melide than at Figino, with maximum rates of 44 and 38 nmol N g^–1^ wet sediment d^–1^, respectively. In slurries amended with ^15^NH_4_^+^ and natural abundance NO_2_^–^, N_2_ production rates were close to 0.01 nmol N g^–1^ wet sediment day^–1^ at both sites, indicating that the contribution of anammox to the total N-removal was <1% (data not shown). Anammox was therefore not further investigated in this study.

**FIGURE 3:**
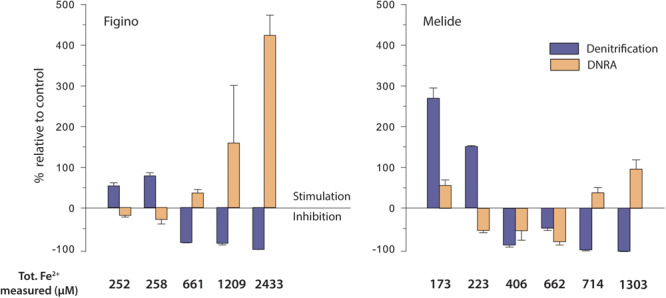
Effect of dissolved ferrous iron on denitrification and DNRA rates in anoxic sediment-biomass incubation experiments amended with ^15^NO_3_^–^ and different Fe^2+^ concentrations. Stimulation (positive values)/inhibition (negative values) is expressed relative to the respective controls without Fe^2+^ additions for each set of experiments (see Supplementary Table 2). Error bars show standard errors (*n* = 3).

### Incubations With Microbial Biomass Only

Incubations with microbial biomass that had been separated from most of the organic and inorganic sedimentary solids were established as a baseline for the treatments amended with inorganic electron donors (Fe^2+^, H_2_S, and Mn^2+^). These experiments were designed to investigate any differential stimulation/inhibition by the tested electron donors under controlled conditions. The removal from sedimentary solids had a major influence on the potential rates and the relative contribution of DNRA to NO_3_^–^ reduction, which decreased from about 31–46% in the regular slurry incubations to <12%. Given the significant deviation from *in situ* conditions, this is not too surprising. In fact, our observation provides putative evidence for nitrate-reduction/partitioning controls other than those that were specifically tested here. More specifically, during the separation step, total particulate organic carbon (POC) was reduced from ∼ 300 to 55 mg L^–1^ (Figino) and ∼ 180 to 25 mg L^–1^ (Melide), while dissolved organic carbon (DOC) concentrations increased from about 510–900 mg L^–1^ and 640–1400 mg L^–1^ for Figino and Melide, respectively. Thus, our findings hint at the importance of organic matter as regulatory factor determining the ratio between denitrification and DNRA (as shown earlier, Nizzoli et al., 2010; Chutivisut et al., 2018), or alternatively suggest that DNRA bacteria are to a larger extent particle-associated. The goal of the present study, however, was to explore the role of inorganic electron donors as potential substrates for anaerobic NO_3_^–^-reduction processes, and their impact on the repartition between denitrification and nitrate ammonification.

### Incubations With Fe^2+^ Additions

In order to investigate the effect of Fe^2+^ on NO_3_^–^ reduction at environmentally relevant concentrations, we conducted incubation experiments with additions of ^15^NO_3_^–^ and various concentrations of dissolved Fe^2+^ (Table 2). While we aimed to lower the ambient Fe^2+^ concentration by separating the microbial biomass from most of the Fe-containing sediment solids and dilution of sediment porewater with Fe-free artificial lake water, total Fe^2+^ background concentrations in the controls were still about 201 and 113 μM at Figino and Melide, respectively, suggesting desorption of Fe^2+^ or dissolution of Fe(II) phases during the separation of microbial biomass. These background Fe^2+^ levels represent 20% of the average ambient Fe^2+^ porewater concentration.

**TABLE 2:** Summary of denitrification, DNRA, NO_3_^–^ consumption rates (all μmol N L^–1^ d^–1^), and the contribution of DNRA to total nitrate reduction (%) in ^15^NO_3_^–^ addition incubation experiments with different Fe^2+^ amendments.

	**Total [Fe^2+^] measured**	**% recovery [Fe^2+^] added**	**Denitrification**	**DNRA**	**NO_3_^–^**	**DNRA contrib.**
	***μM***	***%***		***μmol N L^–1^d^–1^***		***%***

**Figino**	201		5.30(0.65)^*a*^	0.32(0.06)^*a*^	−8.44(1.00)^*a*^	5.7
	252	100	8.18(0.42)*	0.33 (0.02)	−11.07(0.12)*	3.9
	258	63	9.47(0.39)*	0.29 (0.04)	−12.75(0.27)*	3.0
	661	97	0.79(0.03)*	0.20 (0.01)	−3.03(0.18)*	20.2
	1209	100	0.72(0.17)*	0.37 (0.2)	−1.33(0.74)*	33.9
	2433	100	0.01(0.01)*	0.74(0.07)*	−1.00(0.22)*	98.7
**Melide**	113		1.12(0.12)^*a*^	0.10(0.01)^*a*^	−5.29(1.00)^*a*^	8.3
	173	100	4.45(0.29)*	0.19 (0.04)	−5.97(0.58)	4.1
	223	100	3.05(0.03)*	0.13 (0.05)	−3.94(0.13)	4.1
	406	100	0.22(0.06)*	0.08 (0.03)	−0.92(0.18)*	26.7
	662	100	0.70(0.06)*	0.04(0.01)*	−1.42(0.10)*	5.4
	714	56	0.06(0.02)*	0.12 (0.01)	−1.20(0.35)*	66.7
	1303	53	0.03(0.00)*	0.17(0.02)*	−1.33(0.41)*	85

The percentage of recovery was calculated prior to the NO_3_^–^ addition. Positive and negative values correspond to production and consumption, respectively. Standard errors are indicated in parentheses. ^*a*^Average of two different sets of control experiments (*n* = 6). Percentages of stimulation and inhibition of denitrification and DNRA (Figure 3) have been calculated from the respective control for each experiment. *Significantly different (*p* < 0.05) from the respective controls without Fe^2+^ addition for each set of experiments (see Supplementary Table 2).

In all incubation experiments, we observed systematic trends across treatments and between the two sites. At both stations, the lower Fe^2+^ additions (≤258 μM) significantly enhanced N_2_ production, with maximum stimulation relative to the controls of 75 and 250%, at Figino and Melide, respectively (Figure 3). With increasing Fe^2+^ concentrations (≥406 μM), however, N_2_ production decreased to ∼0.01 μmol N L^–1^ d^–1^, indicating almost complete inhibition of denitrification at the highest Fe^2+^ concentration (Figure 3 and Table 2). In contrast, DNRA was partly inhibited by Fe^2+^ at the low and intermediate concentrations, while the highest Fe^2+^ levels (≥2433 and 1303 μM at Figino and Melide, respectively) enhanced DNRA by 408% (Figino) and 88% (Melide) relative to the corresponding controls (Figure 3). Consequently, the relative contribution of DNRA to the total NO_3_^–^ reduction compared to denitrification (i.e., the DEN:DNRA ratio) varied according to the final Fe^2+^ concentration in the incubations. In the controls, DNRA contributed not more than ∼8% to the total nitrate reduction rate. At low Fe^2+^ concentrations, when denitrification was Fe-stimulated the most, the importance of DNRA relative to denitrification decreased (Table 2). In contrast, at Fe^2+^ concentrations higher than 661 μM (Figino) and 406 μM (Melide), the DNRA contribution was ≥20% at both sites (with one exception at 662 μM Fe^2+^ at Melide; Table 2), clearly indicating the differential stimulation/inhibition of DNRA versus denitrification by Fe^2+^.

Consistent with the ^15^N-tracer results, nitrate consumption increased relative to controls when low concentrations of Fe^2+^ were added, particularly at Figino (Table 2). With increasing Fe^2+^ concentrations (i.e., 661 μM and higher), NO_3_^–^ reduction was significantly reduced (Table 2). The nitrate concentration measurements revealed that the net amount of ^15^NO_3_^–^ removed did not match up with the determined products (NO_2_^–^, ^15^N-N_2_ and/or ^15^NH_4_^+^). In most experiments, nitrate consumption was ∼1.3 times higher than the sum of measured ^15^N-labeled products, which may be due to the assimilation or storage uptake by microorganisms and algal cells. The total recovery of dissolved Fe^2+^ added at the beginning of the incubation (after 2–5 days of pre-incubation for pH stabilization) was complete in most treatments (70–100%). But in all experiments, added Fe^2+^ was mostly found in the particulate phase, with only 2–6% remaining in solution. This suggests that Fe^2+^ may have sorbed to surfaces, like remaining sedimentary solids.

### Incubations With H_2_S Additions

Similar to the treatments with Fe^2+^, H_2_S was added at different concentrations to the incubations amended with ^15^NO_3_^–^. In all treatments, denitrification was a more important NO_3_^–^-reducing pathway than DNRA. In control experiments without H_2_S addition, ^15^N-N_2_ production was 8 and 12 times higher than of ^15^NH_4_^+^ at Figino and Melide, respectively (Table 3). Upon addition of H_2_S, the largest fraction of sulfide was removed from solution by reacting with Fe or Mn. Both, the targeted H_2_S (“added”) concentration as well as the actual concentrations of dissolved H_2_S (“measured”) are therefore presented in Table 3. Values reported below represent the targeted concentrations of H_2_S, unless stated otherwise.

**TABLE 3:** Transformation rates of denitrification, DNRA, NO_3_^–^ consumption rates (all μmol N L^–1^ d^–1^), and DNRA contribution to total nitrate reduction (%) from experiments supplemented with ^15^NO_3_^–^ and H_2_S.

	**[H_2_S] added**	**[H_2_S] measured**	**Recovery [H_2_S] added**	**Denitrification**	**DNRA**	**NO_3_**	**DNRA contrib.**
	
	***μM***	***μM***	***%***		***μmol N L^–1^ d^–1^***		***%***
**Figino**	0	0		1.90(0.02)	0.24(0.02)	−5.05(0.24)	11.2
	50	0	0	9.24(0.95)*	0.20(0.02)	−18.34(1.6)*	2.3
	100	0	0	9.84(1.00)*	0.21(0.05)	−23.98(0.44)*	2.0
	500	49	10	6.95(2.31)	0.15(0.07)	−17.48(7.23)	2.1
	1000	125	13	0.07(0.07)*	0.04(0.01)*	−0.22(0.75)*	36.4
**Melide**	0	0.6		1.53(0.40)	0.13(0.04)	−5.69(0.12)	7.8
	50	3	5	7.47(0.62)*	0.09(0.01)	−12.25(3.03)	1.2
	100	1	0	9.35(0.75)*	0.21(0.03)	−20.37(1.57)*	2.2
	500	80	16	17.67(0.33)*	0.17(0.01)	−20.74(3.45)*	1
	1000	132	13	0.32(0.04)	0.06(0.01)	−1.63(0.15)*	15.8

Positive and negative values correspond to production and consumption, respectively, with standard errors given in parenthesis (SE). Concentration of H_2_S and percentage recovery were calculated at T_initial_. *Significantly different from the corresponding control without H_2_S addition (*p* < 0.05).

**FIGURE 4:**
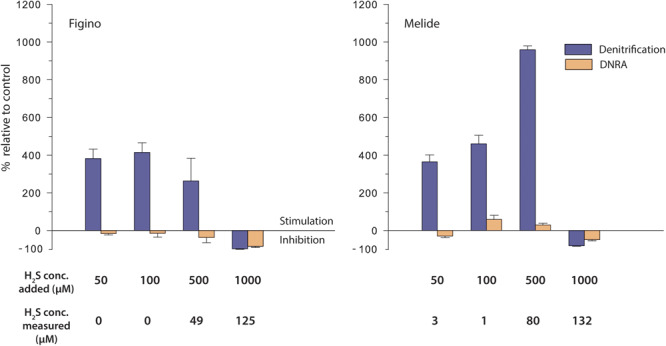
Effect of sulfide on denitrification and DNRA in anoxic sediment biomass incubation experiments amended with ^15^NO_3_^–^ and different H_2_S additions. Stimulation (positive values) / inhibition (negative values) is expressed relative to controls without additions. Error bars represent standard errors (*n* = 3).

Denitrification was significantly enhanced when sulfide was added in the range of 50–500 μM H_2_S (corresponding to ≤80 μM of measured dissolved H_2_S; Table 3). Within this concentration range, denitrification was stimulated in proportion to increasing H_2_S at Melide, while it remained relatively constant at Figino. At 100 and 500 μM H_2_S, the stimulation was highest and reached 414% (Figino) and 959% (Melide), respectively, relative to unamended control incubations (Figure 4). The addition of H_2_S with a targeted concentration of 1 mM, resulted in measured dissolved H_2_S concentrations ≥ 125 μM, and caused a 96 and 80% decline in the denitrification rates at Figino and Melide, respectively (Figure 4). The response of the nitrate ammonifiers to the different H_2_S concentrations was less pronounced and, in most cases, statistically not significant (Table 3). At Figino, ^15^NH_4_^+^ production decreased slightly with increasing H_2_S concentration, while at Melide, DNRA was stimulated by 60 and 30% with 100 and 500 μM H_2_S added, respectively (Figure 4). At both sites, DNRA seemed also to be inhibited at the highest H_2_S levels, although to a lesser extent than denitrification (83 and 47% DNRA decrease at Figino and Melide, respectively).

In incubations exposed to ≤500 μM H_2_S added, the relative contribution of DNRA to the total nitrate reduction decreased to a minimum of ∼2% (Figino) and ∼1% (Melide), respectively (compared to ∼11 and 8% in the controls, respectively). At the highest measured H_2_S concentration, the DNRA contribution increased to about 36% (Figino) and 16% (Melide), suggesting that the balance between denitrification and DNRA is regulated by the environmental H_2_S concentration, as was also the case with Fe^2+^. These experiments were repeated with sediment material collected in March 2017 and displayed very similar patterns (data not shown).

In line with the ^15^N tracer results, NO_3_^–^ consumption increased with increasing H_2_S availability (up to 500 μM H_2_S added; Table 3). As for the other amendment experiments with Fe^2+^, NO_3_^–^ consumption was not balanced by the formation of NO_2_^–^ and ^15^N-N_2_/^15^NH_4_^+^ production. In control incubations, SO_4_^2–^ accumulated at a rate of 1.9 ± 1.8 μmol L^–1^ d^–1^. The production increased with the amount of added H_2_S and reached a maximum rate of 5.9 ± 0.5 μmol L^–1^ d^–1^ in the experiment where 500 μM of H_2_S was added. The rate dropped to 0.3 ± 1.5 μmol L^–1^ d^–1^ at the highest H_2_S concentration tested (Table 3).

### Incubations With Mn^2+^ Additions

**FIGURE 5:**
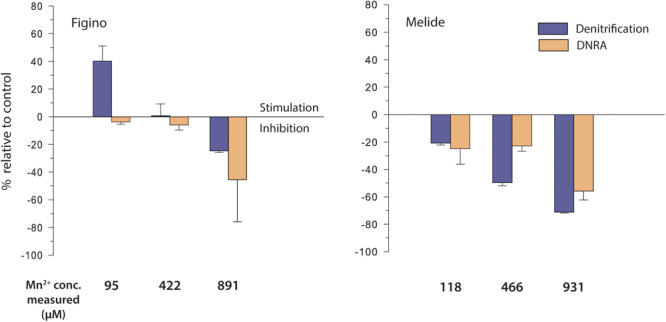
Effect of dissolved manganese on denitrification and DNRA in anoxic sediment biomass incubation experiments amended with ^15^NO_3_^–^ and different Mn^2+^concentrations. Stimulation (positive values) / inhibition (negative values) is expressed relative to controls without additions. Error bars show standard errors (*n* = 3).

As for the Fe^2+^ addition experiments, we aimed to lower the ambient Mn^2+^ concentration in the microbial-biomass separation step (see above), but dissolved Mn^2+^ background concentrations remained substantial, at ∼30 and ∼36 μM for Figino and Melide incubations, respectively. Both in the controls and in all incubations amended with Mn^2+^, production of ^15^N-N_2_ was consistently greater than of ^15^NH_4_^+^. At the Figino station, denitrification and DNRA rates in unamended controls equaled 0.97 ± 0.14 and 0.13 ± 0.03 μmol L^–1^ d^–1^, respectively, and at Melide 1.24 ± 0.09 and 0.06 ± 0.02 μmol L^–1^ d^–1^, respectively. At Figino, at moderate Mn^2+^ addition (95 μM), denitrification was stimulated by about 40% compared to the corresponding controls (Figure 5). At higher Mn^2+^ concentrations, denitrification activity decreased with increasing Mn^2+^ concentration. At Melide, no stimulation of denitrification was observed. Our results revealed a proportional reduction of ^15^N-N_2_ formation with increasing Mn^2+^, corresponding to ∼80% inhibition at the highest Mn^2+^ concentration tested. At both stations, DNRA rates also decreased with rising Mn^2+^ levels.

Transformation rates based on NO_3_^–^ and Mn^2+^ concentration changes were very similar between sites and among treatments. In control incubations, nitrate consumption rates equaled 4.9 ± 0.5 μmol L^–1^ d^–1^ at both stations, and decreased to 2.4 ± 0.4 μmol L^–1^ d^–1^ in incubations supplemented with the highest Mn^2+^ concentration (891 and 931 μM at Figino and Melide, respectively). On the other hand, Mn^2+^ removal rates increased with increasing Mn^2+^ from 0 ± 0.2 μmol L^–1^ d^–1^ in the controls to 3.0 ± 1.6 μmol L^–1^ d^–1^ at highest Mn^2+^ levels. The lack of any significant stimulation of either DNRA or denitrification in most treatments supplemented with Mn^2+^ suggests that Mn^2+^, while being removed from the liquid phase (e.g., through adsorption onto particles), did not play a major role as electron donor for NO_3_^–^ reduction in Lake Lugano sediments.

## Discussion

### Denitrification Versus DNRA in Lake Lugano Sediments

During the sampling campaigns in this study, denitrification was the main N-reduction process in slurries of Lake Lugano (South Basin) sediments. At both stations, the DNRA contribution to the total NO_3_^–^ reduction showed moderate fluctuations between the different seasons, ranging between 31 to 46% but no clear seasonal trend was observed despite of the seasonal variations in bottom water oxygenation and NO_3_^–^ contents. The contribution of DNRA to NO_3_^–^ reduction was significantly higher than observed previously in flow-through whole-core incubations performed with sediments from the same basin (Wenk et al., 2014). Wenk et al. (2014) reported a maximum DNRA contribution to NO_3_^–^ reduction of ∼12%, but also suggested that these measurements should be considered conservative, because they neither accounted for the production of ^14^NH_4_^+^ from ambient background NO_3_^–^ nor for product NH_4_^+^ potentially retained in the intact sediment cores. The use of intact sediment cores provides a more accurate representation of *in situ* N-cycling conditions by maintaining the biogeochemical zonation of the sediment relative to slurry methods (Hansen et al., 2000; Robertson et al., 2019). However, relative potential process rates and their specific regulating factors can better be resolved through controlled manipulation experiments (Robertson et al., 2019), such as those performed here (discussed below).

Similar partitioning of the two dissimilative NO_3_^–^-reducing processes, with a relatively large contribution of DNRA has been observed in a wide range of environments, particularly in reduced sediments with high organic matter content and comparatively low nitrate levels (Burgin and Hamilton, 2007; Dong et al., 2011). The contribution of DNRA to total NO_3_^–^ reduction can be particularly important in both estuarine sediments (∼5–91%; An and Gardner, 2002; Megonigal et al., 2004; Gardner et al., 2006; Burgin and Hamilton, 2007; Dong et al., 2009; Giblin et al., 2013; Roberts et al., 2014; Plummer et al., 2015; Kessler et al., 2019) and freshwater lake sediments (∼3–50%; Nizzoli et al., 2010; Wenk et al., 2014; Robertson and Thamdrup, 2017). Yet, the natural variability in these studied environments is quite high. The OC/NO_3_^–^ ratio is often considered an important regulating factor of the relative contribution of the different benthic NO_3_^–^-reducing processes. DNRA can be stimulated at high OC and low (limiting) NO_3_^–^ levels, while denitrification is generally dominant when NO_3_^–^ is not limiting (Kraft et al., 2014; van den Berg et al., 2016). Differences with respect to the occurrence of DNRA versus denitrification across different ecosystems, however, may also be attributed to variable H_2_S and Fe^2+^ availability, with DNRA presumably being favored over denitrification at high levels of these inorganic substrates.

### Biogeochemical Control of Fe^2+^on Denitrification and DNRA

In order to investigate the effect of inorganic substrates on NO_3_^–^ reduction rates and on the partitioning between denitrification and DNRA we performed incubation experiments with sediment biomass, from which most of the sediment solids have been removed. For the investigation of Fe^2+^, H_2_S and Mn^2+^, which may adsorb to solid phases or react with particle-associated compounds, the exclusion of particulates is helpful in following the consumption of electron donors over time. While the transformation rates in such a system cannot be directly compared to the volume-based biogeochemical rate measurements with natural sediment, these incubations provide insights as to the metabolic potential for stimulation of denitrification and DNRA by alternative electron donors within a carefully controlled system.

The sediments in the southern basin of Lake Lugano are rich in dissolved Fe^2+^ and Mn^2+^ and show seasonal variation in the concentrations of these solutes in the sediment surface layer (Figure 2; Lazzaretti et al., 1992). The apparent relation to fluctuations in bottom water oxygenation and the presence of NO_3_^–^ suggests a potential coupling between NO_3_^–^ reduction and Fe^2+^ oxidation. Our results revealed a complex control from Fe^2+^ on the balance between denitrification and DNRA with relative stimulation of the former and latter at low and high concentrations, respectively. The relative stimulation of DNRA at high Fe^2+^ concentration (≥2433 and 1303 μM Fe^2+^ at Figino and Melide, respectively), agrees qualitatively with observations by Robertson et al. (2016) and Robertson and Thamdrup (2017), who reported a stimulation of DNRA and Fe^2+^ oxidation rates in estuarine and lake sediments with Fe^2+^ additions ranging between 165 and 5000 μM. However, they did not observe a relative stimulation of denitrification at the lower range of Fe^2+^ concentrations tested, which appears to contrast with the present study, where denitrification was favored over DNRA at the lower Fe^2+^ concentrations (≤258 μM) at both stations. Our observations are in line, however, with a pure-culture study by Chakraborty et al. (2011) who also observed enhanced rates of denitrification and increased growth yields in presence of environmentally relevant substrate concentrations (≤250 μM Fe^2+^, 20 μM acetate, 100 μM NO_3_^–^) for mixotrophic *Acidovorax* sp., while at higher Fe^2+^ levels growth ceased as cells became encrusted with Fe^*III*^-oxides. Indeed, *Acidovorax* sp. and *Siderooxydans* sp., another abundant and putative Fe^2+^-oxidizing NO_3_^–^-reducing bacterium, have been detected in the top sediment layers at Figino and Melide using 16S rRNA gene amplicon sequencing (Cojean, 2019). To date, microbial Fe^2+^-dependent NO_3_^–^ reduction is mainly attributed to microbes that sustain their growth energy from the mixotrophic use of Fe^2+^ together with an organic co-substrate (Straub et al., 1996; Melton et al., 2014).

The coupling between Fe^2+^ oxidation and denitrification has also been highlighted in a variety of natural environments including lake waters (Michiels et al., 2017), marine and estuarine sediments (Laufer et al., 2016; Robertson et al., 2016), soils (Ratering and Schnell, 2001), and activated sludge from waste-water treatment plants (Nielsen and Nielsen, 1998). However, the majority of these studies used millimolar substrate concentrations in order to investigate the influence of Fe^2+^ on NO_3_^–^ reduction (Table 1).

In contrast to results from previous studies, it appears that in our experiments, nitrate ammonifiers did not preferentially use Fe^2+^ as electron donor. At low Fe^2+^ levels, there was no indication for any Fe^2+^-induced stimulation of DNRA. In this regard, we argue that the increase in the relative contribution of DNRA to total nitrate reduction at higher Fe^2+^ levels, may not have been a direct result of an increase in the rate of DNRA coupled to Fe^2+^ oxidation. Rather, we speculate that nitrate ammonifiers were less sensitive than denitrifiers to high Fe^2+^ levels, and that very high concentrations of Fe^2+^ may have indirectly favored DNRA bacteria by suppression of organotrophic denitrification and a resulting increased availability of organic substrates.

In previous studies investigating the coupling between Fe^2+^ oxidation and NO_3_^–^ reduction, inhibition of denitrification under Fe-rich conditions has been attributed to cell encrustation from Fe^*III*^-oxide formation around the cell membrane and inside the periplasm (Kappler et al., 2005; Muehe et al., 2009; Klueglein et al., 2014; Nordhoff et al., 2017), which affects bacterial metabolism by limiting substrate uptake, and may even lead to cell damage. However, cell encrustation was, so far, only observed using millimolar Fe^2+^ concentrations whereas our data displayed a significant inhibition of denitrification at around 400 μM Fe^2+^ already, pointing to a potential direct Fe^2+^ toxicity effect on the metabolism. Iron toxicity under anoxic conditions has previously been attributed to inhibition of the F-ATPase (Dunning et al., 1998) and replacement of active-site metal cofactors (Crichton, 2009). But so far, this toxicity effect has only been examined in cultures of anoxygenic phototrophs and streptococci (Dunning et al., 1998; Poulain and Newman, 2009). Our results show that Fe^2+^ inhibition on denitrification in natural habitats may occur at lower Fe^2+^ concentration than previously thought based on studies of pure or enrichment culture experiments.

### Stimulation of Denitrification by H_2_S

In lake sediments, H_2_S is continuously produced by mineralization of sulfur-containing biomass and respiratory SO_4_^2–^ reduction. In ferruginous surface sediments like in the south basin of Lake Lugano, rapid reaction of H_2_S with dissolved Fe^2+^ or Fe-oxides leaves the porewater free of dissolved H_2_S (Lazzaretti et al., 1992). Despite this, independent molecular analyses using 16S rRNA gene sequencing (Cojean, 2019) revealed a variety of sulfur-oxidizing bacteria, among them several abundant taxa with a metabolic potential for anaerobic respiration with nitrate or nitrite (e.g., *Sulfuritalea* sp., *Sulfurimonas* sp., *Sulfurovum* sp. *Thiobacillus* sp.). Moreover, among the three inorganic substrates tested in this study, sulfide additions showed the strongest stimulation on NO_3_^–^ consumption and denitrification.

Stimulation of denitrification by H_2_S has been observed in pure and enrichment cultures (Senga et al., 2006; Campos et al., 2008; Table 1), stratified water columns (Brettar and Rheinheimer, 1991; Burgin et al., 2012; Wenk et al., 2013), sediments (Brunet and Garcia-Gil, 1996; Hayakawa et al., 2013; Deng et al., 2015), but also in engineered systems such as anaerobic digesters (Sher et al., 2008). Often, the degree of H_2_S-induced stimulation was directly related to the H_2_S concentration. In natural systems, denitrification was enhanced primarily at lower H_2_S concentration (<100 μM; e.g., Senga et al., 2006; Burgin et al., 2012; Bowles et al., 2012), while it was strongly inhibited at higher sulfide levels (discussed below). At first sight, denitrification in our study may appear less sensitive toward high H_2_S additions. Considering, however, that most of the added H_2_S (>84%) was removed from solution by reaction with Fe or Mn, we find that our results are in good agreement with these reports. It is likely that increased free H_2_S concentrations ≥ 125 μM (Table 3) in our treatments with highest H_2_S addition inhibited microbial NO_3_^–^ reduction. Uncharged sulfide (H_2_S) can easily diffuse across cell membranes and it is therefore recognized as the most toxic form of the compound (Barton et al., 2014), exerting a stronger inhibitory effect on bacteria than FeS, S^0^ or S_2_O_3_^2–^. At elevated concentrations (>100 μM), free H_2_S can act as growth-inhibitor by e.g., denaturing proteins through disruption of disulfide cross-links between polypeptide chains, or inactivating the redox centers of metalloenzymes, and it can, ultimately lead to cell death (Khan et al., 1990; Wu et al., 2015). In contrast, FeS has been considered as a non-toxic repository of suitable electrons for sulfur bacteria, and inhibition of denitrification by FeS was only observed at >10 mmol S L^–1^ (Garcia-Gil and Golterman, 1993). To date, knowledge on specific effects of H_2_S on NO_3_^–^-reducing microbial groups is scarce. A study on marine microbial communities reports a lag of bacterial growth of potential denitrifiers (*Vibrio* sp., *Marinobacter* sp., *Pseudomonas stutzeri*) with increasing H_2_S concentration (Mirzoyan and Schreier, 2014). Based on pure culture studies reporting on increased concentrations of N-intermediates (NO_2_^–^, NO, N_2_O) in the presence of H_2_S, it has been proposed that H_2_S can have an inhibitory effect on the respective N-compound reducing enzymes (Sørensen et al., 1980; Aelion and Warttinger, 2009). Aside from such conclusions based on the accumulation of N-intermediates, however, knowledge is lacking regarding the mechanisms of inhibition by H_2_S at the enzyme level.

As opposed to denitrification, DNRA is often enhanced under highly sulfidic conditions (Brunet and Garcia-Gil, 1996; Gardner et al., 2006; Lu et al., 2013; Plummer et al., 2015) and may even dominate reductive NO_3_^–^ transformation, leading to the retention of reactive N in the ecosystem (An and Gardner, 2002; Dong et al., 2011; Murphy et al., 2020). Some pure cultures of H_2_S-oxidizing DNRA bacteria can grow at millimolar concentrations of free H_2_S (e.g., Eisenmann et al., 1995), showing that physiological mechanisms exist that allow them to cope with such high levels of this toxic compound. This contrasts with our observations where DNRA activity was somewhat suppressed already in the presence of low H_2_S concentrations, and, just as denitrification, inhibited at free H_2_S concentrations > 125 μM. Unlike previous studies in other environments (e.g., Brunet and Garcia-Gil, 1996; Otte et al., 1999; Sayama et al., 2005) we do not find that H_2_S is the preferred substrate for DNRA bacteria in the ferruginous sediments in the south basin of Lake Lugano where the autochthonous microbial communities are not exposed to significant concentrations of free dissolved H_2_S. We speculate therefore that the selective pressure was not sufficient to enable communities of sulfide-tolerant DNRA bacteria to become enriched. As a consequence, the microbial communities are not well adapted, and thus sensitive, to high H_2_S concentrations. It seems that low free H_2_S concentrations, along with FeS and S^0^ as the most important reduced sulfur species fueling NO_3_^–^ reduction, exert a beneficial remediation effect by increasing the contribution of N-removal (denitrification) relative to N-retention (DNRA).

### Inhibitory Effects of High Mn^2+^ Concentration on Nitrate Reduction

Based on thermodynamic considerations (Luther et al., 1997) and porewater concentration profiles (Aller, 1990; Schulz et al., 1994), Mn^2+^-driven nitrate reduction has been suggested to occur in natural systems, particularly in manganese-rich sediments, but solid experimental proof is still lacking (Madison et al., 2013). The main goal of this part of our work was to assess, whether nitrate-dependent Mn^2+^ oxidation exists in Mn^2+^-rich freshwater sediments. Excluding the Figino Station, where denitrification seemed to be stimulated in the presence of 100 μM Mn^2+^, our results show that rates of both denitrification and DNRA decreased with increasing Mn^2+^ concentrations. No comparable stimulation was observed in sediments from Melide. Based on these results, we conclude that Mn^2+^ does not play a biogeochemically relevant role as reductant for NO_3_^–^ in the sediments under investigation. In line with our data, Schippers et al. (2005) did not find enhanced manganese oxidation with sensitive ^54^Mn radiotracer experiments, in the anoxic water column of the Black Sea when NO_3_^–^, NO_2_^–^, or N_2_O was present. On the other hand, a stimulation of denitrification and DNRA with Mn^2+^ addition was observed in the anoxic water column of the Baltic Sea. It remained unclear, however, whether this stimulation was due to an indirect effect of Mn^2+^, e.g., by the removal of an inhibitory substance (Bonaglia et al., 2016).

In most of our experiments, Mn^2+^ clearly exerted an inhibitory effect on both denitrification and DNRA. The reason for this observation is not clear, but could reflect a general toxicity of high Mn^2+^ concentrations on the activity of microbes. Mn toxicity has been reported for mammals, plants, and prokaryotes (Hohle and O’Brian, 2014), where Mn over-accumulation is often correlated with deficiencies in other transition-metals of physiological relevance. This can then lead to mismetallation of regulatory transcription factors and key enzymes, affecting growth, sensitivity toward reactive oxygen species, and virulence (Zeinert et al., 2018). For instance, Mn can bind to the ferric uptake regulator (Fur) in the Gamma-Proteobacterium *E. coli.* As a consequence, iron import systems are repressed, intracellular Fe levels drop, and, for example, heme synthesis is impeded (Martin et al., 2015). Mn also interferes competitively with secondary Fe import (Martin et al., 2015), and high extracellular Mn^2+^ levels can also competitively inhibit magnesium (Mg^2+^) uptake (Silver and Clark, 1971). Manganese toxicity has been investigated in detail for the soil Alpha-Proteobacterium *Bradyrhizobium japonicum* (Hohle and O’Brian, 2014). It was demonstrated that Mn^2+^ can enter the cells through open Mg^2+^ transporters under low Mg conditions, and exert a toxic effect by displacing Mg in proteins or other macromolecules (Hohle and O’Brian, 2014). Replacing Mg^2+^ with Mn^2+^ as a cofactor in enzymes affects their activity and possibly lead to the disregulation of metabolic pathways (Hohle and O’Brian, 2014).

Whether the mechanisms of Mn^2+^ toxicity established in model organisms also apply to complex environmental communities is not known yet. In our experiments, where we added up to 900 μM MnCl_2_ (in presence of only 0.34 mM Mg^2+^; Supplementary Table 1) and observed a decreasing N-transformation activity with increasing Mn^2+^ levels, Mn^2+^ toxicity by interference with Mg^2+^ homoeostasis is plausible. Yet much more research with environmentally relevant microorganisms is required to better understand the effects of increased transition metal concentrations such as Mn and Fe on environmental microbes and the biogeochemical processes they catalyze.

## Conclusion

Benthic NO_3_^–^ reduction in the South Basin of Lake Lugano is mainly driven by denitrification and DNRA, whereas anammox is negligible. Our results show that Fe^2+^ and H_2_S are important controlling factors for the partitioning between denitrification and DNRA, and that the effect is concentration-dependent. Nitrate was mostly reduced through denitrification at lower levels of dissolved H_2_S (<80 μM) and Fe^2+^ (<258 μM). The relative contribution of DNRA to the overall benthic N reduction increased under highly ferruginous conditions (∼1000 μM Fe^2+^), possibly as a consequence of reduced substrate competition with denitrifiers, which were almost completely inhibited. The role of Mn^2+^ as electron donor for NO_3_^–^ reduction was negligible, as was its influence on the partitioning between denitrification and nitrate ammonification. All three inorganic substrates, however, had strong inhibitory effects at concentrations significantly higher than the prevailing environmental concentrations. Our study implies that the fate of N may be linked to changes in the availability of inorganic substrates within surface sediments.

During periods of bottom water anoxia in eutrophic lakes, reduced inorganic species diffuse out of the sediment into the bottom waters. In the south basin of Lake Lugano, and potentially other iron-rich eutrophic lakes, the zone of Fe^2+^ driven NO_3_^–^ reduction will extend far into the water column. The prevailing conditions of low Fe^2+^ concentrations and nearly absent free H_2_S should be favorable for denitrification rather than DNRA. Periods of extended anoxia under enhanced stratified conditions, however, may lead to the accumulation of significant amounts of Fe^2+^, Mn^2+^, or even H_2_S (Lazzaretti et al., 1992; Lehmann et al., 2015), and the conditions for denitrification and DNRA in near-bottom lake waters or surface sediments may thereby develop from a stimulating into an inhibitory mode, with important implications for the ultimate fate of reactive N in a lake.

We clearly demonstrated the differential role Fe^2+^, H_2_S, and Mn^2+^ can play in regulating the partitioning between denitrification and DNRA. Predictions, however, on how these potential inorganic electron donors act together to possibly shift the balance between the two N-cycling processes and to regulate the overall fixed N-elimination rate in lake sediments remains a challenge.

## Data Availability Statement

The raw data supporting the conclusions of this article will be made available by the authors, without undue reservation.

## Author Contributions

JZ and ML initiated the project. AC performed sample collection and conducted the experimental work. AC, ML, and JZ performed the data analysis and interpretation. AC and JZ prepared the manuscript with input from ML, ER, and BT.

## Conflict of Interest

The authors declare that the research was conducted in the absence of any commercial or financial relationships that could be construed as a potential conflict of interest.
