# Supplementary material for: Controls of H2S, Fe2 +, and Mn2 + on Microbial NO3–-Reducing Processes in Sediments of an Eutrophic Lake
Source: Front Microbiol. 2020 Jun 16;11:1158. doi: 10.3389/fmicb.2020.01158 (PMC7308436; doi:10.3389/fmicb.2020.01158)
Supplement: Supplementary file 1 [file Data_Sheet_1.docx]

Supplementary Material

**Supplementary Table 1.** Composition of artificial lake water used for incubation experiments. Synthetic lake water was prepared according to Smith et al., (2002) to emulate the major ion composition of Lake Lugano water, devoid of dissolved nitrogen compounds.

**Supplementary Table 2.** Overview of the different experiments performed in this study.

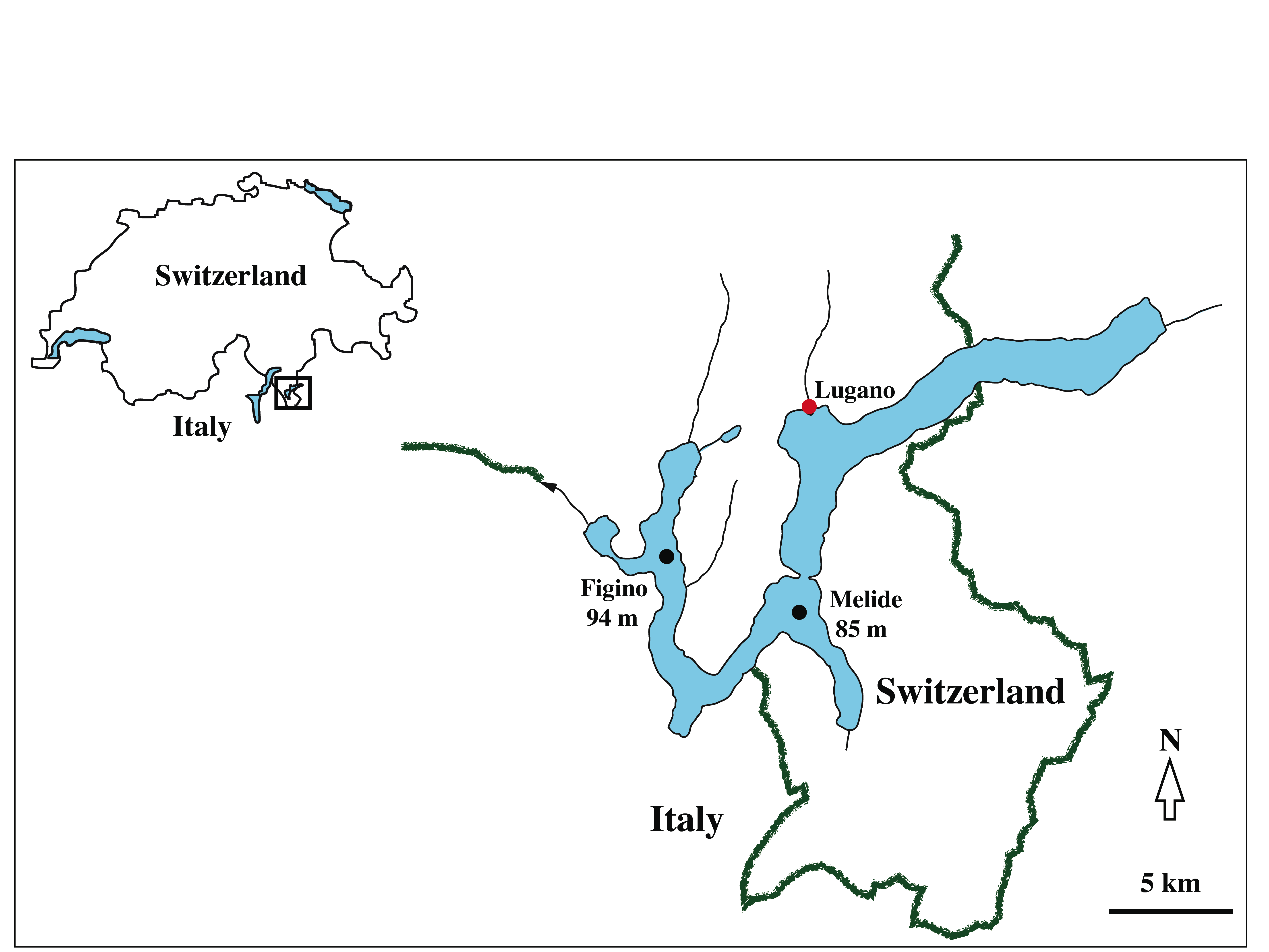


**Supplementary Figure 1.** Map of Lake Lugano and the two sampling sites Figino and Melide.


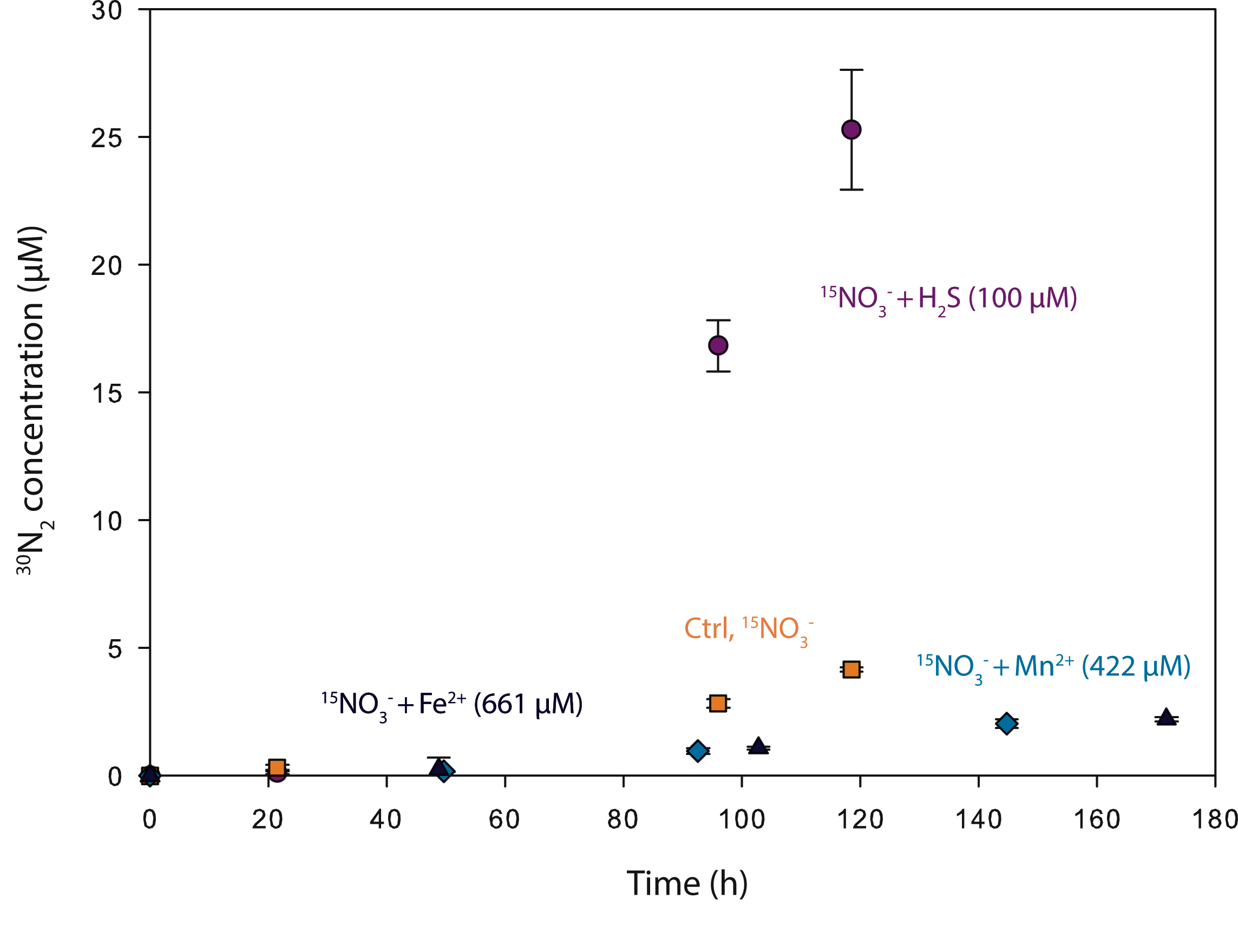
**Supplementary Figure 2.** Exemplary times-series data of ^30^N_2_ concentrations during denitrification incubation experiments with microbial biomass and additions of Fe^2+^, H_2_S and Mn^2+^ at Figino. Error bars correspond to standard error of triplicate experiments.
